# Occupational and environmental asbestos exposure and the risk of lung cancer in Korea: A case-control study in South Chungcheong Province of Korea

**DOI:** 10.1371/journal.pone.0249790

**Published:** 2021-04-08

**Authors:** Da-An Huh, Min-Sung Kang, Jiyun Lee, Ji Yoon Choi, Kyong Whan Moon, Yong-Jin Lee

**Affiliations:** 1 Department of Health Science, Graduate School at Korea University, Seoul, Republic of Korea; 2 Asbestos Environmental Health Center, Soonchunhyang University Cheonan Hospital, Cheonan-si, Chungcheongnam-do, Republic of Korea; 3 Department of Health and Safety Convergence Science, Graduate School at Korea University, Seoul, Republic of Korea; 4 BK21 FOUR R&E Center for Learning Health System & Department of Health and Environmental Science, Graduate School at Korea University, Seoul, Republic of Korea; 5 Department of Occupational & Environmental Medicine, Soonchunhyang University, Cheonan-si, Chungcheongnam-do, Republic of Korea; Universidade Federal do Rio Grande - FURG, BRAZIL

## Abstract

Despite the use of large amounts of asbestos in the 1990s, few studies have been conducted in Korea on occupational and environmental asbestos exposure and lung cancer risk. The main aim of this study was to estimate the risk of lung cancer development caused by occupational and environmental asbestos exposures in residents of South Chungcheong Province, where about half of the asbestos mines in Korea operated. We conducted a case-control study, for which the information on asbestos exposure history and demographic characteristics was provided by the Environmental Health Center for asbestos of Soonchunhyang University Cheonan Hospital. After adjusting for all covariates, the odds ratios for lung cancer tended to increase with higher exposure probability for both occupational as well as environmental asbestos. The relative risk of occupational asbestos exposure was higher than that of environmental exposure; the interaction of co-exposure was not statistically significant. The estimated means of the latency period were significantly shorter in participants who were engaged in the production of asbestos-containing products and in those who lived near asbestos industries as compared to other groups.

## Introduction

Lung cancer is the most common cancer worldwide. Approximately 2 million people worldwide are diagnosed with lung cancer annually, of which 1.76 million die [1]. The death toll from lung cancer in Korea in 2018 accounted for approximately 22.5% of all cancer deaths [2], and the proportion is expected to rise to approximately 29% in 2020 [3]. Although various factors such as smoking and air pollution are known risk factors for lung cancer, occupational asbestos exposure is also a leading cause of asbestos-related lung cancer in Korea [4].

The production of asbestos in Korea began in the mid-1930s [5], and the amount of its use increased steadily until the mid-1990s to reach a peak in 1996 [6, 7]. Of the 38 operational asbestos mines in Korea, 25 were present in South Chungcheong Province [8, 9]. It has been reported that about 1,100 workers and 2,000 residents had been involved in the production of 190,379 tons of asbestos from 1954 to 1986 in a Gwangcheon mine located in South Chungcheong Province [9, 10]. All asbestos mines in Korea were closed sequentially until 1984, but, until recently, asbestos had been detected in the soil around abandoned mines [11]. Therefore, although much of the occupational asbestos exposure may have occurred during the period of operation of the mine, it can be expected that environmental asbestos exposure has continued from the mine’s operational period till the present day.

To provide relief to the victims of asbestos exposure, the Ministry of Environment of Korea enacted the Asbestos Injury Relief Act in 2011 [12]. The purpose of this Act was to redress the damage to health caused by asbestos by seeking measures to pay benefits to asbestos-inflicted disease sufferers and their bereaved family members [12]. In addition, the Ministry of Environment has designated two Environmental Health Centers for Asbestos to operate a health surveillance system to identify asbestos victims. In South Chungcheong Province, where asbestos mines were concentrated, during the period from 2009–2018 when the centers were operational, about 1,500 asbestos victims were found.

The relief system focuses more on finding asbestos victims and rescuing them from health damage rather than researching on the health effects of asbestos exposure characteristics in Korea. Therefore, although the government is collecting a lot of information about exposure history and demographic characteristics of victims through the relief system, little research has been conducted in Korea on occupational and environmental asbestos exposure and lung cancer risk. It is necessary to investigate the health effects of asbestos considering its sources of exposure, duration of exposure, and individual characteristics for effective treatment of asbestos-exposed groups.

## Materials and methods

### Study population

In this study, we used the information collected by the Soonchunhyang University Cheonan Hospital, one of the Environmental Health Centers for Asbestos in Korea [13]. We conducted a case-control study to identify the association of asbestos exposure with the risk of lung cancer. Residents over 20 years of age who have lived in South Chungcheong Province for more than 5 years and who received a chest radiography, or a computed tomographic scan were recruited.

Among these residents, 184 patients with newly diagnosed primary lung cancer between 2009 and 2018 were potentially eligible cases. All the cases included were histologically identified by a panel of pathologists. We excluded 5 patients whose asbestos exposure history was unclear, and 179 patients were finally selected as cases. The controls were negative for lung cancer selected from the 21,832 people of the same population receiving a chest radiography or a computed tomographic scan. Participants were excluded from controls if they were diagnosed with other asbestos-related lung diseases such as malignant mesothelioma and asbestosis. Five controls per case were selected randomly from the same group [14] after frequency matching for the 3-year age group, sex, and region of residence to adjust for the effects of relevant confounders.

### Asbestos exposure history of the participants

In 2009, a structured questionnaire was developed to collect the information of the study subjects. To validate the questionnaire, asbestos experts who understand our research topic were asked to review the questionnaire. Researchers improved the questionnaire every January by correcting the issues that occurred in the previous year. Information on lifetime asbestos exposure was collected using questionnaires by researchers who participated in the development of the questionnaire. Occupational exposure to asbestos was defined as a history of occupational contact with asbestos fibers for at least one year. The information on asbestos exposure included the name of the workplace, type of the job, duration of work, and age at first exposure. To reduce recall bias, we also used past records such as their certificate of employment collected by the Environmental Health Center for Asbestos to verify the location and operating period of the workplace provided by the participants. The types of occupations were classified into three categories: extraction work (asbestos extraction, conveyance, and grinding), production of asbestos-containing products (cements, slates, and fabrics), and maintenance work (demolition and repair of asbestos-containing buildings or equipment).

Environmental asbestos exposure was defined as a history of non-occupational contact with asbestos fibers caused by sources of airborne asbestos which included those from asbestos mines, industries, and loading spaces. Detailed information collected included the region of residence, type of exposure sources, distance from the sources, duration of residence, age at first exposure, and history of soil cultivation. To verify the accuracy of the exposure information provided by the participants, we used data of Korea’s past exposure sources of asbestos collected by the Environmental Health Center for Asbestos and the participants’ residential registration documents.

We also set the criteria to assess the probability of asbestos exposure from occupational and environmental sources by modifying the methods of previous studies [15, 16]. The probability of exposure was categorized into six stages: not exposed, possible, probable, likely, definite, and unknown. The criteria for distinguishing probabilities were defined separately for occupational and environmental exposures. The duration of work and the distance from exposure sources to residences were used, respectively, to set off criteria. Detailed definitions are shown in Table 1.

**Table 1:** Definition of categories of probability for occupational and environmental exposure to asbestos.

Probability	Definition
Occupational asbestos exposure	
Not exposed	A person who did not experience any occupational asbestos exposure; a person who worked in asbestos-related jobs for less than 1 year (<1 y)
Possible	Working period of more than 1 year and less than 5 years (1–5 y)
Probable	Working period of more than 5 years and less than 10 years (5–10 y)
Likely	Working period of more than 10 years and less than 30 years (10–30 y)
Definite	Working period of more than 30 years (≥30 y)
Unknown	Lack of information to determine exposure
Environmental asbestos exposure	
Not exposed	A person who did not experience any occupational asbestos exposure; a person who lived more than 5 km away from the exposure sources (>5 km)
Possible	Over 2 km and not more than 5 km distance from the exposure sources (2–5 km)
Probable	Over 1 km and not more than 2 km distance from the exposure sources (1–2 km)
Likely	Over 0.5 km and not more than 1 km distance from the exposure sources (0.5–1 km)
Definite	Not more than 0.5 km distance from the exposure sources (≤0.5 km)
Unknown	Lack of information to determine exposure

The latency periods between initial exposure and diagnosis of lung cancer were calculated in the case group based on the survey results. For patients who experienced occupational asbestos exposure, the age at which they started working was considered as the initial exposure time. On the other hand, for patients who experienced environmental exposure, the year in which the exposure sources began to operate or the year in which they began to live near the exposure sources was considered as the initial exposure time.

### Statistical analysis

Odds ratios (ORs) and 95% confidence intervals (CIs) were estimated using unconditional logistic regression [17, 18]. The estimates were calculated for each exposure category and compared to the unexposed group (reference). We developed a sequence of three models to identify the influence of potential confounders: crude; adjusted for age, sex, education level, and pack-year (model 1); and, further adjusted for probability of exposure (model 2). Age and pack-year were considered continuous variables. Education levels were classified as less than high school, high school graduation, and college or more.

The joint effect of occupational and environmental exposure on lung cancer was examined after adjusting for all covariates. We categorized exposure probability into dichotomous variables and combined these to classify them into the following 4 groups: no and no (reference), no and yes, yes and no, yes and yes. In addition, we defined the participants whose exposure probability was classified as Likely or Definite as “high” and compared the risk of “high” and “high” group to the reference group. The relative excess risk due to interaction (RERI) was calculated, and we also computed 95% CIs for the RERI following the standard delta method based on a Taylor series expansion [19].

The Student t-test and analysis of covariance (ANCOVA) were used to evaluate differences in the mean latency period between groups. The age- or full-adjusted mean of latency periods for each group were estimated using ANCOVA.

All statistical analyses were performed using SPSS version 26.0 (IBM, New York, NY, USA), and p<0.05 was considered statistically significant.

### Ethics approval

The institutional review board of Soonchunhyang University Cheonan Hospital approved the collection and utilization of data for this study (2009-04-001). All subjects were informed by researchers face-to-face about all aspects of this study, and they gave their informed consent documents for inclusion before they participated in the study.

## Results

Table 2 shows the descriptive statistics of 179 cases and 895 controls. About 84% of the participants were male in both cases and controls, and the mean age was similar in both groups at 78.94 and 78.88 years, respectively. The proportion of lung cancer cases in current smokers was higher than that in the never smoked group, and the cases group had a higher mean pack-year. The number of participants who experienced occupational or environmental exposure were 223 and 971, respectively, and 191 were with co-exposure. The proportion of cases in the occupational exposure group was 35%, which was approximately twice that of the environmental exposure group.

**Table 2:** Descriptive statistics of total cases and controls participating in the study (191 participants are co-exposed to occupational and environmental asbestos).

Variables	Total		Background level		Occupational exposure		Environmental exposure	
	Cases	Controls	Cases	Controls	Cases	Controls	Cases	Controls
	n = 179 (%)	n = 895 (%)	n = 3 (%)	n = 68 (%)	n = 77 (%)	n = 146 (%)	n = 161 (%)	n = 810 (%)
Age (mean ± SD)	78.94 ± 8.43	78.88 ± 8.43	78.00 ± 4.00	75.94 ± 9.66	80.54 ± 7.76	80.89 ± 8.89	79.11 ± 7.75	79.19 ± 8.31
Gender								
Male	151 (20.0)	755 (80.0)	3 (5.0)	57 (95)	73 (34.4)	139 (65.6)	113 (16.3)	681 (83.7)
Female	28 (20.0)	140 (80.0)	0 (0.0)	11 (100)	4 (36.4)	7 (63.6)	28 (17.8)	129 (82.2)
Education level								
< High school	60 (8.6)	635 (91.4)	0 (0.0)	38 (100.0)	31 (21.4)	114 (78.6)	56 (8.8)	582 (91.2)
High school	8 (8.6)	85 (91.4)	0 (0.0)	15 (100.0)	2 (28.6)	5 (71.4)	8 (10.5)	68 (89.5)
> High school	2 (5.6)	34 (94.4)	0 (0.0)	12 (100.0)	0 (0.0)	1 (100.0)	2 (8.3)	22 (91.7)
Unknown	109 (43.6)	141 (56.4)	3 (50.0)	3 (50.0)	44 (62.9)	26 (37.1)	95 (40.8)	138 (59.2)
Smoking status								
Never smoked	44 (11.1)	351 (88.9)	0 (0.0)	26 (100.0)	13 (22.4)	45 (77.6)	43 (11.8)	320 (88.2)
Past smoker	70 (16.7)	350 (83.3)	1 (3.2)	30 (96.8)	36 (39.1)	56 (60.9)	62 (16.5)	314 (83.5)
Current smoker	65 (25.1)	194 (74.9)	2 (14.3)	12 (85.7)	28 (38.4)	45 (61.6)	56 (24.1)	176 (75.9)
Pack-year (mean ± SD)	23.36 ± 22.89	20.88 ± 21.83	24.67 ± 9.50	17.71 ± 18.44	29.68 ± 26.12	24.00 ± 23.05	21.86 ± 22.37	21.11 ± 22.11

The risk of lung cancer according to occupational asbestos exposure is shown in Table 3. The OR in the exposed group was 3.08 (95% CI: 1.86, 5.11) compared to the unexposed group in the fully adjusted model. The highest OR was observed in workers who were engaged in the production of asbestos-containing products such as cements, slates, and fabrics (OR = 8.70; 95% CI: 3.13, 24.18). The OR was positively associated with higher exposure probability and tended to decrease in groups with higher initial exposure age.

**Table 3:** Risk of lung cancer according to occupational asbestos exposure.

Variables	Cases	Controls	Crude OR^c^ (CI^d^)	Model 1^a^ OR^c^ (CI^d^)	Model 2^b^ OR^c^ (CI^d^)
	n (%)	n (%)			
Occupational exposure					
No	102 (12.0)	749 (88.0)	Ref.	Ref.	Ref.
Yes	77 (34.5)	146 (65.5)	3.87 (2.74, 5.47)	3.91 (2.51, 6.09)	3.08 (1.86, 5.11)
Work site					
Extraction work^e^	48 (28.7)	119 (71.3)	2.96 (2.00, 4.39)	3.20 (1.94, 5.27)	2.26 (1.27, 4.00)
Production work^f^	23 (52.3)	21 (47.7)	8.04 (4.30, 15.05)	7.10 (3.12, 16.16)	8.70 (3.13, 24.18)
Maintenance work^g^	6 (50.0)	6 (50.0)	7.34 (2.32, 23.20)	5.21 (1.22, 22.25)	5.11 (1.11, 23.58)
Exposure probability					
Possible	26 (28.0)	67 (72.0)	2.85 (1.73, 4.69)	2.89 (1.53, 5.44)	2.01 (0.97, 4.17)
Probable	11 (20.8)	42 (79.2)	1.92 (0.96, 3.86)	2.96 (1.34, 6.57)	2.44 (0.98, 6.11)
Likely	29 (53.7)	25 (46.3)	8.52 (4.80, 15.12)	8.24 (3.91, 17.33)	7.48 (3.07, 18.21)
Definite	9 (64.3)	5 (35.7)	13.22 (4.35, 40.21)	7.97 (2.31, 27.43)	11.25 (2.54, 49.82)
Unknown	2 (22.2)	7 (77.8)	2.10 (0.43, 10.24)	1.12 (0.20, 6.38)	0.62 (0.10, 3.97)
Age at first exposure (y)					
<20	19 (46.3)	22 (53.7)	6.34 (3.32, 12.12)	8.77 (3.94, 19.50)	6.79 (2.64, 17.45)
20–29	20 (34.5)	38 (65.5)	3.87 (2.17, 6.90)	6.75 (3.25, 14.03)	6.69 (2.85, 15.68)
30–39	14 (51.9)	13 (48.1)	7.91 (3.62, 17.30)	7.57 (2.84, 20.15)	4.34 (1.35, 13.96)
≥40	11 (57.9)	8 (42.1)	10.10 (3.97, 25.69)	4.83 (1.34, 17.45)	5.15 (0.95, 27.85)
Unknown	13 (16.7)	65 (83.3)	1.47 (0.78, 2.86)	1.11 (0.52, 2.36)	0.78 (0.32, 1.87)

^a^ Model 1 was adjusted for age, sex, education level, and pack-year.

^b^ Model 2 was adjusted for all variables included in model 1 and was further adjusted for the probability of occupational exposure.

^c^ Odds ratio.

^d^ Confidence interval.

^e^ Extraction work included asbestos extraction, conveyance, and grinding.

^f^ Production work included production of asbestos-containing products such as cements, slates, and fabric.

^g^ Maintenance work included demolition and repair of asbestos-containing buildings or equipment.

Table 4 presents the risk of lung cancer according to environmental asbestos exposure. The OR did not significantly increase in the exposed group, but tended to increase with a higher exposure probability after adjusting for all covariates. In particular, for the group with exposure probability classified as Definite, the OR was 6.21 times that of the unexposed participants (OR = 6.21; 95% CI: 1.61, 24.02). No significant trend was observed in the association between age at first exposure to asbestos and lung cancer. The increased OR was significantly higher in the participants involved in soil cultivation than in the non-experienced group.

**Table 4:** Risk of lung cancer according to environmental asbestos exposure.

Variables	Cases	Controls	Crude OR^c^ (CI^d^)	Model 1^a^ OR^c^ (CI^d^)	Model 2^b^ OR^c^ (CI^d^)
	n (%)	n (%)			
Environmental exposure					
No	3 (4.2)	68 (95.8)	Ref.	Ref.	Ref.
Yes	161 (16.6)	810 (83.4)	4.51 (1.40, 14.50)	2.64 (0.77, 9.04)	1.03 (0.38, 2.77)
Type of exposure source					
Asbestos mines	135 (15.8)	722 (84.2)	4.24 (1.31, 13.67)	2.90 (0.84, 9.96)	2.10 (0.61, 7.26)
Asbestos industries	17 (42.5)	23 (57.5)	16.75 (4.50, 62.42)	2.59 (0.62, 10.87)	1.74 (0.40, 7.57)
Loading space	9 (12.2)	65 (87.8)	3.14 (0.81, 12.11)	0.76 (0.15, 3.86)	0.48 (0.09, 2.54)
Exposure probability					
Possible	2 (3.1)	62 (96.9)	0.73 (0.12, 4.52)	0.22 (0.03, 1.52)	0.19 (0.03, 1.27)
Probable	17 (4.3)	383 (95.8)	1.01 (0.29, 3.53)	0.71 (0.19, 2.70)	0.53 (0.14, 2.04)
Likely	103 (24.5)	318 (75.5)	7.34 (2.26, 23.83)	4.87 (1.39, 17.08)	3.53 (1.00, 12.51)
Definite	39 (45.3)	47 (54.7)	18.81 (5.49, 64.46)	7.68 (2.01, 29.29)	6.21 (1.61, 24.02)
Age at first exposure (y)					
<20	43 (11.6)	328 (88.4)	2.97 (0.90, 9.86)	2.42 (0.67, 8.71)	1.57 (0.43, 5.74)
20–29	38 (25.3)	112 (74.7)	7.69 (2.29, 25.88)	6.83 (1.84, 25.40)	4.56 (1.21, 17.1)
30–39	41 (19.3)	171 (80.7)	5.44 (1.63, 18.14)	3.45 (0.95, 12.54)	2.28 (0.62, 8.36)
≥40	19 (11.4)	148 (88.6)	2.91 (0.83, 10.17)	0.70 (0.17, 2.80)	0.45 (0.11, 1.87)
Unknown	20 (28.2)	51 (71.8)	8.89 (2.51, 31.55)	2.78 (0.70, 11.07)	2.63 (0.66, 10.47)
Experience of cultivation					
No	27 (19.7)	110 (80.3)	5.56 (1.63, 19.04)	1.09 (0.28, 4.25)	0.86 (0.22, 3.39)
Yes	62 (36.9)	106 (63.1))	13.26 (4.00, 43.93)	5.65 (1.52, 20.97)	4.47 (1.20, 16.69)
Unknown	74 (10.8)	611 (89.2)	2.75 (0.84, 8.95)	2.77 (0.78, 9.82)	1.80 (0.50, 6.42)

^a^ Model 1 was adjusted for age, sex, education level, and pack-year.

^b^ Model 2 was adjusted for all variables included in model 1 and was further adjusted for the probability of environmental exposure.

^c^ Odds ratio.

^d^ Confidence interval.

Table 5 shows the results of estimating the individual and joint effects of occupational and environmental exposure on lung cancer. The relative risk of occupational asbestos exposure was higher than that of environmental exposure. The OR for participants with both high occupational and environmental exposure probabilities was 20.56 (95% CI: 4.44, 95.21) compared to the reference group after adjusting for all covariates. This is larger than the additive effect of high occupational exposure only (OR = 12.84; 95% CI: 2.84, 58.11) and high environmental exposure only (OR = 1.84; 95% CI: 0.53, 6.38); however, the interaction on the additive scale was not statistically significant (RERI = 22.77; p = 0.374).

**Table 5:** Risk of lung cancer according to combined occupational and environmental exposure to asbestos.

Category of co-exposure	Cases	Controls	OR^a^
	n (%)	n (%)	estimate^b^ (95% CI^c^)
Occupational & environmental			
No and No	3 (4.2)	68 (95.8)	Ref.
No and Yes	99 (12.7)	681 (87.3)	1.84 (0.53, 6.38)
Yes and No	15 (46.9)	17 (53.1)	12.84 (2.84, 58.11)
Yes^d^ and Yes^d^	47 (28.7)	117 (71.3)	5.13 (1.42, 18.56)
High^e^ and High^e^	15 (55.6)	12 (44.4)	20.56 (4.44, 95.21)

^a^ Odds ratio.

^b^ The model was adjusted for age, sex, education level, and pack-year.

^c^ Confidence interval.

^d^ Combination of occupational and environmental exposures excluding high and high.

^e^ The participants whose exposure probability was classified as Likely or Definite were defined as “High.”.

Measure of interaction on the additive scale: RERI (95% CI) = 22.77 (-27.40, 72.94); p = 0.374.

The adjusted mean latency periods according to the characteristics of the participants are shown in Table 6. After controlling all covariates, the latency period for participants with occupational asbestos exposure was shorter than that for the environmental exposure and co-exposure groups, but the difference was not statistically significant. However, the workers who engaged in the production of asbestos-containing products and the residents who lived near asbestos industries had significantly shorter latency periods than other groups. For smokers, the latency periods tended to decrease with higher pack-years.

**Table 6:** Adjusted mean latency periods according to the characteristics of the participants.

Variables	Adjusted for age		Full adjusted^a^	
	Estimate (95% CI^b^)	p-value	Estimate (95% CI^b^)	p-value
Exposure type				
Occupational exposure	39.06 (32.06, 46.06)	0.058	38.99 (29.87, 48.11)	0.123
Environmental exposure	46.95 (44.30, 49.60)		46.57 (40.77, 52.37)	
Co-exposure	48.42 (45.24, 51.60)		46.48 (39.91, 53.04)	
Type of job				
Extraction work^c^	49.80 (46.40, 53.20)	0.026	45.22 (36.54, 53.90)	0.035
Production work^d^	36.89 (27.85, 45.93)		33.18 (21.32, 45.04)	
Maintenance work^e^	45.65 (40.65, 50.64)		43.23 (35.03, 51.43)	
Type of exposure source				
Asbestos mines	48.83 (46.70, 50.96)	0.011	46.90 (41.33, 52.48)	0.045
Asbestos industries	39.25 (31.45, 47.05)		39.29 (29.24, 49.34)	
Loading space	41.80 (35.77, 47.83)		40.48 (32.21, 48.75)	
Gender				
Male	46.58 (44.47, 48.69)	0.392	42.79 (37.37, 48.21)	0.440
Female	49.04 (43.77, 54.32)		45.23 (37.01, 53.46)	
Smoking status				
Never smoked	48.63 (44.44, 52.82)	0.658	43.92 (37.01, 50.84)	0.960
Past smoker	46.53 (43.22, 49.84)		44.74 (37.58, 51.90)	
Current smoker	46.33 (42.73, 49.93)		44.78 (37.25, 52.30)	
Pack-year				
<10	49.82 (46.30, 53.34)	<0.001	47.11 (39.98, 54.23)	0.006
10–30	47.49 (42.91, 52.07)		45.33 (38.86, 51.80)	
30–50	49.63 (45.88, 53.37)		46.31 (38.12, 54.50)	
≥50	48.58 (43.03, 54.13)		44.98 (36.89, 53.08)	
Unknown	38.23 (34.09, 42.37)		36.33 (28.90, 43.77)	

^a^ The model was adjusted for age, sex, education level, pack-year, and exposure type.

^b^ Confidence interval.

^c^ Extraction work included asbestos extraction, conveyance, and grinding.

^d^ Production work included production of asbestos-containing products such as cements, slates, and fabrics.

^e^ Maintenance work included demolition and repair of asbestos-containing buildings or equipment.

## Discussion

The aim of this study was to verify our hypothesis that occupational and environmental exposure to asbestos could increase the risk of lung cancer development. We conducted a case-control study in the South Chungcheong Province of Korea. A total of 179 cases and 895 controls were selected, and surveys were conducted on the participants’ exposure history and demographic characteristics. After adjusting for all covariates, the ORs for lung cancer tended to increase with a higher exposure probability in both occupational and environmental asbestos groups. The relative risk of occupational asbestos exposure was higher than that of environmental exposure, and the interaction of co-exposure was not statistically significant. The estimated means of the latency period were significantly shorter in participants who engaged in the production of asbestos-containing products and who lived near asbestos industries than in the other groups.

Asbestos had been imported in Korea since the 1960s, and it decreased after reaching its maximum in 1992 [20]. About 82% of the imported asbestos was used in the 1990s to produce slates and thermal insulation materials [5]. Previous studies had predicted that the mortality from asbestos-related diseases in Korea would peak in the 2020s, considering the amount of asbestos used and the latency periods [21, 22]. To provide relief to these asbestos victims, the Ministry of Environment enacted the Asbestos Injury Relief Act in 2011 [12]. However, because the law focuses on identifying and rescuing victims, little research had been conducted on the effects of asbestos exposure, especially the health effects of environmental asbestos exposure. Some reports from the Ministry of Environment suggest that the rate of lung cancer caused by asbestos exposure is about five times higher than that in without-exposure groups of similar age, but there is insufficient evidence [23]. It is necessary to assess the health effects of asbestos exposure according to regions and exposure sources because the risk of asbestos exposure may differ depending on living habits, technology, legislation, and attitude toward the risk [24]. As the beginning of this, our study was conducted in South Chungcheong Province, where about half of the asbestos mines used to operate previously. In addition to South Chungcheong Province, South Gyeongsang, North Chungcheong, and Gangwon Province were considered as the presumed asbestos exposure areas where many environmental sources of exposure existed such as asbestos slate buildings and asbestos factories. Currently, two environmental health centers continue to conduct health impact surveys on residents in these areas [13], and further studies on the risk of asbestos in each area should be carried out using these data in the future.

The results of this study were similar to those of previous studies except that the risks of occupational and environmental exposures were estimated to be slightly higher [25–27]. Gustavsson et al. reported that excess risk of lung cancer at low exposure levels of asbestos associated with a RR of 1.9 (95% CI 1.32–2.74) [26], and Pohlabeln et al. presented that the estimated OR of asbestos at the occupational exposure level was 1.18 (95% CI 1.05–1.32) [27]. This is because the mean age of the subjects in this study was about 79 years, which was higher than that in previous studies, and those who experienced at least one year of occupational exposure or who lived near the exposure sources for at least 10 years were included as subjects in the study. The result that the risk increased with longer exposure periods and shorter distance from the exposure source was similar to that obtained in a previous study [16].

We found that people who worked in factories producing asbestos-containing products had a higher risk of lung cancer than those who worked in asbestos extraction or maintenance work such as demolition and repair of asbestos-containing buildings or equipment. In addition, patients with lung cancer who worked at or lived near the factories had a shorter latency period than other groups. Because different types of asbestos could have different exposure responses [28], we are of the opinion that this difference is caused by the type of asbestos to which the person is exposed. The most common type of asbestos mined in Korea was chrysotile; therefore, it can be assumed that the type of asbestos encountered by workers involved in asbestos extraction was chrysotile [29]. However, historical records show that crocidolite or amosite was imported into the factory to produce specific products [30], and about 10% of asbestos-containing building materials contained crocidolite or amosite [31]. A recent retrospective cohort study also reported that 43% workers at asbestos textile factories were exposed to crocidolite [32]. Therefore, patients with lung cancer associated with asbestos factories are estimated to have a high rate of exposure to crocidolite or amosite, but further study is required.

In 2015, when the Ministry of Environment examined the soil around two abandoned mines in South Chungcheong Province, an asbestos concentration of more than 1% was detected in the soil. This asbestos was detected in an area of about 20,000 m2 that included farmlands and forest lands. This means that residents of that area had been exposed to environmental asbestos since the 1990s when the mines were closed. This also explains why the OR of participants in our study who were involved in cultivation near asbestos exposure sources was 4.47 times higher than that of the reference group. The Korean government is conducting soil restoration if more than 1% concentration of asbestos is detected in the soil; however, a way to prevent the scattering of asbestos from the sources of exposure is needed.

Several limitations of the present study must be considered. Because of the lack of information, we used only probability, and not intensity, to assess the exposure of asbestos. Especially in the case of occupational exposure, information was limited in our study because there was insufficient data on the mean asbestos concentrations at each work site. However, given the limited information about the actual asbestos exposure levels in many previous studies, the exposure probability using the exposure duration and the distance from exposure sources can be used as valid proxy indicators to estimate exposure [33]. In addition, participants may have given exaggerated information about their exposure because of their awareness of the purpose of the study. However, this situation would have occurred in both cases and controls. These non-differential biases move the estimated risk to null; the actual risk may be much greater than we estimated.

## Conclusions

This is the first case-control study to assess the effects of asbestos exposure on lung cancer in South Korea. Our results are consistent with those of previous studies that showed that occupational and environmental asbestos exposure was related to lung cancer development. The risk of lung cancer was positively associated with an increase in the probability of asbestos exposure. In addition, the estimated means of the latency period were significantly shorter in participants who were engaged in the production of asbestos-containing products and who lived near asbestos industries than in other groups.

## Supporting information

S1 Table Questionnaire (in Korean). (PDF)
