# Supplementary material for: Occupational and environmental asbestos exposure and the risk of lung cancer in Korea: A case-control study in South Chungcheong Province of Korea
Source: PLoS One. 2021 Apr 8;16(4):e0249790. doi: 10.1371/journal.pone.0249790 (PMC8031370; doi:10.1371/journal.pone.0249790)
Supplement: S1 Table — (PDF) [file pone.0249790.s001.pdf]

석면건강영향조사 설문지

성　명		검진일자	201　년　월　일	검진번호	
주민등록번호	________ - ________			성별	□ 남　□ 여
키	cm	몸무게	Kg	체질량지수	
집 전화		휴대전화		e-mail	
주　소					
가족 연락처	(관계 :　　)	집 전화		휴대전화	

일반적 특성

■ 음주력

아래 문항을 읽고 해당되는 번호에 표시 또는 내용을 작성하여 주십시오.

1-1. 일주일에 평균 며칠이나 술을 마십니까?

① 마시지 않음　② 1일　③ 2일　④ 3일　⑤ 4일　⑥ 5일　⑦ 6일　⑧ 일주일 내내

1-2. 술을 드실 때 보통 하루에 얼마나 마십니까? (술 종류에 관계없이) ________ 잔/1일

■ 흡연력

2. 아래 문항을 읽고 자신의 현재 상태에 해당되는 내용을 작성하여 주십시오.

☞ 지금까지 평생 총 5갑(100개비)이상의 담배를 피운 적이 있습니까?

① 아니오		
② 예, 지금은 끊었음 (과거 흡연)	⇨ 금연 전까지 담배를 몇 년 이나 피우셨습니까?	총　년
	⇨ 금연하기 전 하루 평균 흡연량은 몇 개피 였습니까?	개피/1일
	⇨ 현재까지의 금연기간은 얼마 동안입니까 ?	총　년
③ 예, 현재도 흡연 중 (현재 흡연)	⇨ 몇 년 째 담배를 피우고 계십니까?	총　년
	⇨ 하루 평균 흡연량은 몇 개피 였습니까?	개피/1일

■ 신체활동력

3-1. 최근 일주일간 평소보다 숨이 훨씬 더 차게 만드는 격렬한 활동을 하루 20분 이상 시행한 날은 며칠이었습니까? (예 : 달리기, 에어로빅, 빠른 속도로 자전거 타기, 등산 등)

① 없었음　② 1일　③ 2일　④ 3일　⑤ 4일　⑥ 5일　⑦ 6일　⑧ 일주일 내내

3-2. 최근 일주일간 평소보다 숨이 조금 더 차게 만드는 중간정도 활동을, 하루 30분 이상 시행한 날은 며칠이었습니까? (예 : 빠르게 걷기, 복식 테니스치기, 보통 속도로 자전거타기, 엎드려 걸레질하기 등)
※ 3-1에서 응답한 신체활동은 제외

① 없었음　② 1일　③ 2일　④ 3일　⑤ 4일　⑥ 5일　⑦ 6일　⑧ 일주일 내내

3-3. 최근 일주일간 한 번에 적어도 10분 이상씩 걸은 경우를 모두 합하여, 하루 총 30분 이상 걸은 날은 며칠이었습니까? (예 : 가벼운 운동, 출퇴근이나 여가 시간에 걷기 포함)
※ 3-1, 3-2에서 응답한 신체활동은 제외

① 없었음　② 1일　③ 2일　④ 3일　⑤ 4일　⑥ 5일　⑦ 6일　⑧ 일주일 내내

●

질병력 및 암 질환

아래 항목에 있는 호흡기 질환 및 암을 본인 및 가족 중에서 앓았거나, 앓고 계신 경우 해당 내용에 응답하여 주십시오. [해당되는 곳에 모두 ○ 를 ●로 표시하시오.]

호흡기 질환	본인	가족	암(악성종양)	본인	가족
1.진폐증(석면폐증)	○	○ (관계 :)	1. 폐암	○	○ (관계 :)
2. 폐섬유화증/ 간질성폐질환	○	○ (관계 :)	2. 악성중피종	○	○ (관계 :)
3. 흉막질환 (늑막염 포함)	○	○ (관계 :)	3. 기관지암	○	○ (관계 :)
4. 결핵	○	○ (관계 :)	4. 후두암	○	○ (관계 :)
5. 폐성심 (심장비대)	○	○ (관계 :)	5. 난소암	○	○ (관계 :)
6. 기관지 확장증	○	○ (관계 :)	6. 장암 (대장/직장암)	○	○ (관계 :)
7. 만성 기관지염	○	○ (관계 :)	7. 위암	○	○ (관계 :)
8. 기관지천식	○	○ (관계 :)			
9. 폐기종	○	○ (관계 :)			
10. 기타(________)	○	○ (관계 :)			

자각증상(본인)

1. 귀하는 지난 1년 동안 아래의 호흡기 증상이 지속적으로 2주 이상 있었던 적이 있었습니까?

증상	아니오	예
마른기침	①	②
가래동반 기침	①	②
호흡곤란	①	②
객혈	①	②
흉통(가슴통증)	①	②

●

환경 중 석면 노출

1. 귀하가 태어나서 지금까지 실제 거주하신 곳의 주소를 모두 적어주십시오.
(현재 거주지 부터 과거 태어난 거주지의 순서대로 적어주십시오.)

번호	주 소					거주 기간 (예 : 1956년~ 1960년)
	광역시/ (도)	시/군/구	읍/면/동	리 / 마을	번지	
1						~ 현재까지
2						~
3						~
4						~
5						~
6						~

2. 태어나서 지금까지 다음과 같은 석면 노출 사항이 있으면 해당되는 모든 문항의 □에 ■로 체크하여 주시고 거주 기간을 작성하여 주시기 바랍니다.

석면 노출	거주 기간 (1956년 2월 ~1960년 8월)
□ 폐석면 광산 등의 석면더미에서 놀았던 경험이 있다.	~
□ 석면을 가지고 놀았던 경험이 있다.	~
□ 슬레이트 지붕 또는 축사를 수리한 적이 있다.	~
□ 집에서 석면을 사용하여 일을 하거나 석면제품을 수리한 적이 있다.	~
□ 석면광산 2km 이내에서 농사를 한 적이 있다.	~
□ 집 2km 이내에 석면광산이 있었다.	~
□ 집 2km 이내에 석면공장이 있었다.	~
□ 집 2km 이내에 재개발 또는 재건축으로 인한 건축물 철거가 있었다.	~
□ 집 2km 이내에 자동차 정비소(공업사)가 있었다.	~
□ 집 2km 이내에 선박제조 또는 수리업체가 있었다.	~
□ 집 2km 이내에 공단이 있었다.	~

●

석면 노출 작업

■ <표 1> 아래 항목은 석면관련 직업에 대한 질문입니다. 참고하여 설문에 응답해주시기 바랍니다.

구분	번호	석면관련 직업	석면 작업내용 및 생산품
석면광산	1.	석면광산업	석면광산에서 석면을 채취하는 업무
	2.	석면광산관련 작업	석면광산에서 생산된 석면을 가공하는 작업(분쇄, 포장 등)
석면 함유 제품 생산	3.	석면시멘트제품 제조	슬레이트, 천정재, 밤라이트, 석면압출제품 등 생산
	4.	석면마찰재	브레이크 라이닝, 브레이크 패드, 면클러치 판, 석면특수브레이크 생산
	5.	석면 조인트시트	가스켓 생산
	6.	석면 방직(방적)제품	석면사, 석면로프, 석면사 패킹, 석면테이프, 석면 포, 석면장갑 생산
석면/ 석면 함유 제품 사용	7.	건축업	슬레이트 지붕 및 건축물 철거, 개보수 작업, 인테리어
	8.	건설업	전기작업 및 천정텍스, 타일, 보일러 설치 등 석면 함유 제품 사용
	9.	철강/화학공장/벽돌 제조 등 산업	배관 설치, 배관 정비, 배관 해체
	10.	배관 설치 및 정비작업	(철강/화학공장/벽돌제조) 이외의 건설 등 각종 산업에서 석면제품 사용
	11.	자동차. 철도 정비작업	브레이크 수리 및 정비, 하체작업
	12.	선박제조 및 수리업	정비, 용접작업, 석면포 사용작업
	13.	발전소 및 고열작업	발전소, 주물공장 등 고열작업에 포함된 석면제품 사용
	14.	기타 석면제품	석면함유 모터 정류자, 산업용 석면단열재 등
	15.	석면 가내수공업	석면물레질, 가정에서 석면작업
	16.	군대에서 석면취급	석면건축물, 석면제품 취급, 정비
기타	17.	기타 석면 노출원	상세히 기술( )

직업 중 석면 노출 (본인)

1. 귀하(본인)가 일하면서 석면을 취급하거나 노출 되었습니까 ?

① 아니오 ➪ (직업 중 석면 노출(동거가족)으로)

② 예 ➪ (2번으로)

2. 평생동안 귀하(본인)가 종사하였던 석면관련 직업을 위의 <표 1> 에서 모두 고르시고, <u>가장 오랜 기간 근무한</u> 순서대로 기입해 주시기 바랍니다.

	직업	근무 시작 (연령)			근무 마지막 (연령)			석면관련 직업번호	구체적인 작업내용
		년	월	연령	년	월	연령		
예시	본인 ●	1975	03	26	1984	10	35	2	석면 분쇄 및 운반
1	본인 ○								
2	본인 ○								
3	본인 ○								
4	본인 ○								

●

동거가족에 의한 석면 노출

1. 귀하(본인)와 같이 살았던 가족(배우자, 부모 등)으로부터 석면노출이 있었습니까 ?

① 아니오

② 예 ⇨ (2번으로)

2. 귀하(본인)에게 석면을 노출시킨 가족의 직무(일)를 위의 <표 1> 에서 모두 고르시고, 가족관계와 함께 해당 직무를 가장 오랜 기간 근무한 순서대로 기입해 주시기 바랍니다.

	직업	근무 시작 (연령)			근무 마지막 (연령)			석면관련 직업번호	구체적인 작업내용
		년	월	연령	년	월	연령		
예시	가족 ● (관계: 부)	1962	06	43	1970	10	51	7	슬레이트 지붕 개보수
1	( )								
2	( )								
3	( )								
4	( )								

3. 설문, 문진 및 진찰결과 석면폐질환과 관련하여 수검자와 가족에 대한 중요한 소견이 있는 경우에는 상세히 기술하여 주십시오(상담의사가 작성).

4. 수검자 설문 시 석면폐질환과 관련하여 수검자와 가족에 대한 중요한 소견이 있는 경우에는 상세히 기술하여 주십시오(조사원이 작성).

상담의사 : (서명)
